# Supplementary material for: An Updated Overview of MRI Injuries in Neonatal Encephalopathy: LyTONEPAL Cohort
Source: Children (Basel). 2022 Apr 14;9(4):561. doi: 10.3390/children9040561 (PMC9032533; doi:10.3390/children9040561)
Supplement: Supplementary file 1 [file children-09-00561-s001.zip › children-1644211-supplementary.pdf]

CHU DE GRENOBLE

# CRF LyTONEPAL

**Version 4.0, 14/09/2015**

***Long Term Outcome of Neonatal hypoxic EncePhALopathy in the era of neuroprotective treatment with hypothermia***

## *Cerebral MRI*

MRI completed: □ yes □ no

If yes:

- Date of completion: |__|__| / |__|__| / |__|__|__|__| (dd/mm/yyyy)

- Sequence:
  - T1 □ yes □ no
  - T2 □ yes □ no
  - Diffusion □ yes □ no
  - T2* or gradient echo □ yes □ no
  - Other: □ yes □ no

    If yes for Other, please specify: ______________________

- Image quality:
  - □ Very good (without any artefacts)
  - □ Good (at least the 3 sequences: axial T1, axial T2 and axial good quality Diffusion)
  - □ Poor/ not interpretable (at least one out of the 3 sequences above present artifacts)

- Is the MRI normal: □yes □no

  **If the MRI is normal (= yes), proceed to the conclusion question (degree of injury)**

  **If the MRI is ABNORMAL (= no)**:
  - Degree of certainty for this abnormality:

    □ no doubt □ moderate doubt □ significant doubt

**If the MRI is abnormal: Ask the questions: degree of certainty and DETECTED ANOMALIES SECTION**

**DETECTED ANOMALIES**

**1. Basal Ganglia anomalies: □ yes □ no**

If "no" for the basal ganglia anomalies, proceed to the anomalies of posterior limb internal capsule (PLIC) question.

If « yes » for basal ganglia anomalies:

- Degree of certainty: □ no doubt □ moderate doubt □ significant doubt
- Diffuse: □ yes □ no
- Localized: □ yes □ no

If « yes » for basal ganglia anomalies, location of the anomaly:

- Putamen: □ yes □ no If yes: □ right □ left □ right and left
- Globus pallidus: □ yes □ no If yes: □ right □ left □ right and left
- Thalamus: □ yes □ no If yes: □ right □ left □ right and left
- Caudal nucleus: □ yes □ no If yes: □ right □ left □ right and left et gauche

If « yes » for basal ganglia anomalies, sequences where the anomaly is visualized:

- T1: □ yes □ no If yes: □ hyper signal □ iso signal □ hypo signal
- T2: □ yes □ no If yes: □ hyper signal □ iso signal □ hypo signal
- Diffusion: □ yes □ no If yes: □ restriction □ increase

**2. Posterior limb internal capsule anomalies (PLIC):** □ **yes** □ **no**

If « no » for posterior limb international capsule anomalies, proceed to the cortical anomalies question

If « yes » for posterior limb internal capsule anomalies:

- Degree of certainty: □ no doubt □ moderate doubt □ significant doubt

If « yes » for posterior limb internal capsule anomalies:

- Unilateral: □ yes □ no If yes: □ right □ left
- Bilateral: □ yes □ no

If « yes » for posterior limb internal capsule anomalies, <u>sequences that visualize the anomaly</u>:

- T1: □ yes □ no If yes: □ iso signal □ hypo signal (according to Basal ganglia)
- T2: □ yes □ no
- Diffusion: □ yes □no

**3. Cortical anomalies:** □ **yes** □ **no**

If "no" for cortical anomalies, proceed to the white matter anomalies question

If « yes » for cortical anomalies:

- Degree of certainty: □ no doubt □ moderate doubt □ significant doubt

If "yes" for cortical anomalies:

- Diffuse: □ yes □ no
- Localized: □ yes □ no

If "yes" for cortical anomalies: <u>Location:</u>

- Rolandic: □ yes □no If yes: □ right □ left □ right and left
- Insular: □ yes □no If yes: □ right □ left □ right and left
- Mesial temporal: □ yes □no If yes: □ right □ left □ right and left
- Occipital: □ yes □no If yes: □ right □ left □ right and left

- Anterior junctional: □ yes □ no If yes: □ right □ left □ right and left
- Posterior junctional: □ yes □ no If yes: □ right □ left □ right and left

If "yes" for cortical anomalies: Visibility

- T1: □ yes □ no If yes: □ hypersignal □ iso signal □ hypo signal
- T2: □ yes □ no If yes: □ hypersignal □ iso signal □ hypo signal
- Diffusion: □ yes □ no If yes: □ restriction □ increase

**4. White matter anomalies: □ yes □ no**

If "no" for white matter anomalies, proceed to the corpus callosum question

If "yes" for white matter anomalies:

- Degree of certainty: □ no doubt □ moderate doubt □ significant doubt

If « yes » for white matter anomalies:

- Diffuse: □ yes □ no
- Localized: □ yes □ no

  If yes for localized: punctiform (<5mm): □ yes □ no

  If yes for punctiform: number: □ ≤ 5 or □ >5

  If yes for localized: areas: □ yes □ no

If yes for white matter anomalies, Location:

- Periventricular: □ yes □ no If yes: □ right □ left □ right and left
- Anterior junctional: □ yes □ no If yes: □ right □ left □ right and left
- Posterior junctional: □ yes □ no If yes: □ right □ left □ right and left
- Centrum semiovale: □ yes □ no If yes: □ right □ left □ right and left
- Sub-cortical: □ yes □ no

If « yes » for sub cortical location, specify by answering the following questions:

- Frontal: □ yes □ no If yes: □ right □ left □ right and left
- Temporal: □ yes □ no If yes: □ right □ left □ right and left
- Parietal: □ yes □ no If yes: □ right □ left □ right and left
- Occipital: □ yes □ no If yes: □ right □ left □ right and left

If « yes » for white matter, sequences visualizing the anomaly:

- T1 □ yes □ no If yes: □ hypersignal □ hypo signal
- T2 □ yes □ no If yes: □ hypersignal □ hypo signal

- Diffusion □ yes □ no If yes: □ restriction □ increase

5. **Corpus callosum anomalies:** □ **yes** □ **no**

If no for corpus callosum anomalies, proceed to the brainstem anomaly question

If yes for the corpus callosum anomalies

- Degree of certainty: □ no doubt □ moderate doubt □ significant doubt

If yes for corpus callosum anomalies, Location:

- Splenium: □ yes □ no
- Knee: □ yes □ no

If yes for corpus callosum anomalies, sequences visualizing the anomaly:

- T1: □ yes □ no If yes: □ hypersignal □ hypo signal
- T2: □ yes □ no If yes: □ hypersignal □ hypo signal
- Diffusion: □ yes □ no If yes: □ restriction □ increase

6. **Brainstem anomalies:** □ **yes** □ **no**

If no for brainstem anomalies, proceed to the cerebellum anomalies question

If yes for the brainstem anomalies:

- Degree of certainty: □ no doubt □ moderate doubt □ significant doubt

If yes for brainstem anomalies:

- Diffuse: □ yes □ no
- Localized: □ yes □ no

If yes for brainstem anomalies, Location:

- Pons (dorsal): □ yes □ no
- Midbrain: □ yes □ no
- Bulb: □ yes □ no

If yes for brainstem anomalies, sequences visualizing the anomaly:

- T1: □ yes □ no If yes: □ hyper signal □ hypo signal
- T2: □ yes □ no If yes: □ hyper signal □ hypo signal
- Diffusion: □ yes □ no If yes: □ restriction □ increase

7. **Cerebellum anomalies** □ **yes** □ **no**

If no for cerebellum anomalies, proceed to the « others » question: hemorrhagic injuries

If yes for cerebellum anomalies:

- Degree of certainty: □ no doubt □ moderate doubt □ significant doubt

If yes for cerebellum anomalies:

- Diffuse: □ yes □ no
- Localized: □ yes □ no

If yes for cerebellum anomalies, Location:

- Vermis: □ yes □ no
- Hemisphere: □ yes □ no If yes: □ right □ left □ right and left

**8. Others: Hemorrhagic injuries: □yes □no**

If no for hemorrhagic injuries, proceed to the conclusion

If yes for hemorrhagic injuries

- Degree of certainty: □ no doubt □ moderate doubt □ significant doubt

If yes for hemorrhagic injuries

- Intra parenchymal: □ yes □ no
- Intra ventricular: □ yes □ no
- Extra axial: □ yes □ no

**Conclusion**

- Degree of injury:

□ normal examination □ moderate injury □ severe injury

If the examination is normal, proceed to the open comments section

If injuries (severe or moderate), precise the site and/or extension:

- Peripheral (cortical, subcortical or junctional) □ yes □ no
- Central (Basal ganglia thalami) □ yes □ no
- Diffuse □ yes □ no

Open comment: (malformation injury, other)

______________________________________________________________________________

__________________________________________________
